# Transcriptional profiling of feline infectious peritonitis virus infection in CRFK cells and in PBMCs from FIP diagnosed cats

**DOI:** 10.1186/1743-422X-10-329

**Published:** 2013-11-09

**Authors:** Mohammad Syamsul Reza Harun, Choong Oi Kuan, Gayathri Thevi Selvarajah, Tan Sheau Wei, Siti Suri Arshad, Mohd Hair Bejo, Abdul Rahman Omar

**Affiliations:** 1 Laboratory of Vaccines and Immunotherapeutics, Institute of Bioscience, Universiti Putra Malaysia, 43400, UPM Serdang, Selangor, Malaysia; 2 Infectomics Cluster, Advanced Medical & Dental Institute, Universiti Sains Malaysia, Bertam, 13200 Kepala Batas, Pulau Pinang, Malaysia; 3 Department of Veterinary Pathology & Microbiology, Faculty of Veterinary Medicine, Universiti Putra Malaysia, 43400 UPM Serdang, Selangor, Malaysia; 4 Department of Veterinary Clinical Studies, Faculty of Veterinary Medicine, Universiti Putra Malaysia, 43400 UPM Serdang, Selangor, Malaysia

**Keywords:** FIPV, CRFK, PBMCs, Transcriptome, RT-qPCR, Gene expression, Fold change

## Abstract

**Background:**

Feline Infectious Peritonitis (FIP) is a lethal systemic disease, caused by the FIP Virus (FIPV); a virulent mutant of Feline Enteric Coronavirus (FECV). Currently, the viruses virulence determinants and host gene expressions during FIPV infection are not fully understood.

**Methods:**

RNA sequencing of Crandell Rees Feline Kidney (CRFK) cells, infected with FIPV strain 79–1146 at 3 hours post infection (h.p.i), were sequenced using the Illumina next generation sequencing approach. Bioinformatic’s analysis, based on *Felis catus* 2X annotated shotgun reference genome, using CLC bio Genome Workbench mapped both control and infected cell reads to 18899 genes out of 19046 annotated genes. Kal’s Z test statistical analysis was used to analyse the differentially expressed genes from the infected CRFK cells. Real time RT-qPCR was developed for further transcriptional profiling of three genes (PD-1, PD-L1 and A3H) in infected CRFK cells and Peripheral Blood Mononuclear Cells (PBMCs) from healthy and FIP-diseased cats.

**Results:**

Based on Kal’s Z-test, with False Discovery Rate (FDR) <0.05 and >1.99 fold change on gene expressions, a total of 61 genes were differentially expressed by both samples, where 44 genes were up-regulated and the remainder were down-regulated. Most genes were closely clustered together, suggesting a homogeneous expression. The majority of the genes that were significantly regulated, were those associated with monocytes-macrophage and Th1 cell functions, and the regulation of apoptosis. Real time RT-qPCR developed focusing on 2 up-regulated genes (PD-L1 and A3H) together with an apoptosis associated gene PD-1 expressions in FIPV infected CRFK cells and in PBMCs from healthy and FIP diagnosed cats produced concordant results with transcriptome data.

**Conclusion:**

The possible roles of these genes, and their importance in feline coronaviruses infection, are discussed.

## Background

Feline coronaviruses are enveloped, positive sense RNA viruses that can be classified into two biotypes, namely low virulent Feline Enteric Coronavirus (FECV) and highly virulent Feline Infectious Peritonitis Virus (FIPV). FECV is very common in the cat population worldwide, and has been shown to have infected 20-60% pet cats and shed by 75-100% cats in multi-cat environments [1,2]. Of those shedding the virus, 1-5% will develop Feline Infectious Peritonitis (FIP) disease [3]. Within the biotypes, the viruses are differentiated further into serotype I and serotype II, based on virus neutralizing antibodies. Type I FECV and FIPV strains are more ubiquitous worldwide and are more likely to cause clinical FIP, while type II strains are less common, but more adaptable to cell culture [2].

It has been suggested that FIPV, the causative agent for FIP, is a mutant form of FECV [4,5]; where several possible nature of mutation responsible for the increase in virulence has been characterized. Studies have shown that several mutations throughout the FIPV genome were detected, but mutations at 3c membrane protein and 7b secretory glycoprotein genes were suggested to be responsible for transforming FECV to FIPV [4,5]. A recent study revealed that mutation of the S1/S2 locus and modulation of a furin recognition site normally present in the S gene of FECVs is a critical contributing factor for development of FIP [6]. Furthermore, it was found that FIPV infection is associated with T cell depletion by apoptosis; although the virus cannot infect CD4+ and CD8+ T cells [7,8]. Therefore, apoptosis of CD4+ and CD8+ T cells is probably caused by mediators from infected macrophages and/or intestinal epithelial cells [8,9]. Hence, little is known about the interaction of the virus and host cells; especially the early cellular transcriptional responses towards virus infection, virus mechanism of inducing T cell apoptosis, and the absence of cell-mediated immunity (CMI) response in FIP infected cats.

The use of a next generation sequencing approach in RNA sequencing has facilitated understanding in defining the expression profiles of cellular responses during pathogen infections. This method has been proven to be helpful in explaining the pathogenesis of various viruses [10,11], including Feline Immunodeficiency Virus (FIV) infection [12,13]. Furthermore, the availability of complete 1.9X of cat genome, using the Whole Genome Shotgun (WGS) approach, provides valuable information for the bioinformatic’s analysis of feline host responses, following pathogen infection. Moreover, the cat genome contigs were aligned, mapped, and annotated to NCBI annotated genome sequence of six index mammalian genomes (human, chimpanzee, mouse, rat, dog, and cow) using MegaBLAST [14].

Previous study has shown that more than 70% of FIPV strain 79–1146 were internalized by CRFK cells at 3 hours post infection [15]. Hence, in this study, mRNA from CRFK cells infected with FIPV strain 79–1146 at 3 h.p.i were sequenced using Illumina next generation sequencing technology. The generated data was then analyzed using CLC bio Genomic Workbench, where the genes were compared to *Felis catus* 1.9X annotated shotgun reference genome. Kal’s Z-test on expression proportions [16] was used to determine significantly expressed genes. Genes expressed with a False Discovery Rate (FDR) <0.05 and >1.99 fold change were considered for further analysis.

## Results

### Early gene expression of FIPV infected CRFK cells

Overall, the trimmed sequence reads match to 25,689 annotated transcripts; where only 215 (0.8%) were statistically significant (Kal’s Z test, *p* < 0.05), and out of the significant matched, only 96 (44.7%) transcripts were expressed with fold absolute change of 2 or more. Of these 96 transcripts, 76 (79.2%) were up-regulated and the remainder were down-regulated. After BLAST analysis, 76 up-regulated transcripts were matched to 44 genes while 20 down-regulated transcripts were matched to 17 genes. Of the 44 up-regulated genes, there were 2 transcripts per gene for 32 genes, but only one transcript per gene for the remaining 12 genes. Meanwhile, of the 17 down-regulated genes, there was one transcript per gene for 14 genes and 2 transcripts per gene for 3 genes.

**Figure 1:**
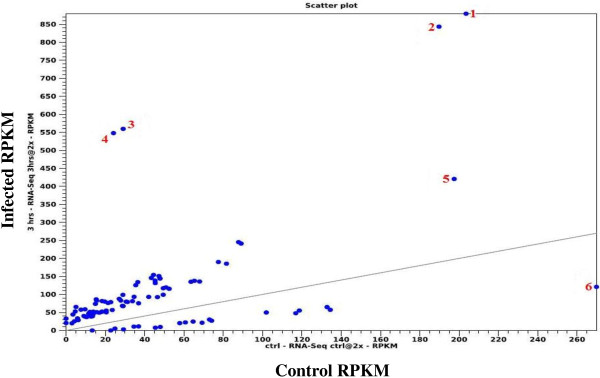
**Scatter plot of control RNA-seq RPKM versus infected RNA-seq RPKM of significant transcripts (p < 0.05) with absolute fold change value of 2 or more.** Most genes from both samples are closely clustered except for 4 genes label 1 to 6. 1 and 2 are MX1 transcripts 1 and 2, respectively, 3 and 4 = RSAD2 transcripts 1 and 2, respectively, 5 = PLIN2 and 6 = SERPINB1.

As shown in Figure 1, the RPKM of control samples was plotted against the RPKM of infected samples; and as such, genes with equal expression will line-up on the diagonal identity line while genes with different expression values will either be over or under the diagonal line. The further away the point is from the identity line, the larger is the difference between its expressions in one experiment compared with the other. Except for 4 genes (MX1, RSAD2, PLIN2, and SERPINB1), most genes from both samples were closely clustered together, thus suggesting a homogeneous expression. The plot excluded 3 genes (CCL8, RNF7, and RPL39) that had infinite fold change expressions.

Table 1 shows the top 20 up-regulated genes (in decreasing order) and their functions. The majority of the genes were those associated with immune response, while the remainder were associated with apoptosis, cell cycle, cytokine, and ubiquitination activities. Furthermore, there were also 5 Interferon Stimulated Genes (ISGs) coded for 6 proteins (RSAD2, A3C, A3H, MDA5, IFI35, and MX1) that were involved in inhibiting viral entry, replication, and production. Interestingly, one gene (PD-L1), which negatively regulates immune response in viral infection, was also found to be highly up-regulated. Meanwhile, the majority of the down-regulated genes were involved in pro-inflammatory cytokine’s activation, CMI, and anti-apoptosis activities (Table 2). Two unique down-regulated genes (RNF7 and RPL39) were found to be expressed in control uninfected cells only, where the former had anti-apoptotic effect and the latter translated RNA to protein.

**Table 1:** List of top 20 up regulated genes and their functions

**No.**	**Fold change**	**BLAST result**	**Accession number**	**Gene product function**
1**	∞/ ∞	CCL8	S 67956	induce Th cytokinesattract monocyte, lymhpocyte, NK cell, dendritic cell, basophil & eosinophil
2*	22.8/ 19.33	RSAD2	XM_ 002921192	ISG, inhibit viral protein & RNA synthesis
3*	12.98/ 7.64	CXCL10	XM_ 002924730	induce Th1 cells response stimulate monocyte, NK & T cell migration
4*	12.73/ 11.36	SLAMF7	XM_ 002928442	stimulate NK cell cytotoxicity, B cell growth, promote lymphocytes adhesion
**5**	6.68	FSTL3	XM_ 850129	regulate cell to cell adhesion
6*	6.11/ 2.34	ATF3	XM_ 847382	promote apoptosis and cell proliferation, promotes Th1 and NK cells activity
7*	6.04/ 4.68	MFSD2A	AL 663070	regulate cell growth, cell adhesion & motility
**8**	5.76	ESE1	XM_ 002914595	regulate inflammation & cell differentiation
9**	5.65/ 4.07	A3C & A3H	EU 109281	ISG, antiviral cytidine deaminase, edit viral RNA/DNA causing mutation
10**	5.35/ 4.48	MDA5	NG_ 011495	ISG, viral RNA sensor induce cytokines and interferons
11*	4.96/ 3.00	IFI35	XM_ 002930054	ISG, inhibit viral gene transcription
12*	4.46/ 3.90	TRIM25	XM_ 548223	ubiquitination of RIG-I and ISG15 induce type I IFN
13*	4.46/ 4.34	MX1	NM_ 001003134	ISG, induce apoptosis in virus infected cells
14**	4.24/ 3.29	PD-L1	EU 246348	negative regulation of immune response, induce IL-10
15*	4.10/ 3.31	PHF11	AL 139321	Th1 cytokines activation
16**	3.98/ 3.54	RUNTX1	NG_ 011402	regulate gene transcription for T cell differentiation and function
17**	3.79/2.85	BHLHE40	XM_ 002919988	regulate gene transcription, lymphocyte activation & cell cycle and cell death
18*	3.69/ 3.56	HERC5	XM_ 002913599	ubiquitination of ISG15 and RIG-I
**19**	3.69	CCL17	NM_ 001009849	activate T cell development and maturation
**20**	3.65	DTX3L	XM_ 002927235	protect cell from DNA damage

*Two transcripts were found from the same gene.

**Two transcripts from the same gene with similar E-value, score and gaps.

**Table 2:** List of all 17 down regulated genes and their functions

**No.**	**Fold change**	**BLAST result**	**Accession number**	**Gene Pproduct punction**
**01**	-2.04	JAG1	NG_ 007496	TLRs response, positive regulation of Notch signaling pathway
**02**	-2.04	c-KIT	NM_ 001009837	signal transduction, apoptosis inducer, clathrin dependent endocytosis
03**	-2.14/ -2.44	JUB	XM_ 537368	co-trancriptional repressor with GFI-1, cell adhesion
**04**	-2.22	SERPINB1	AF 053630	neutrophil proteolytic activity inhibitor,
**05**	-2.34	CD59	NM_ 001112709	T-cell differentiation, gene transcription repression
**06**	-2.40	COTL1	XM_ 001144958	pro-inflammatory leukotrienes activation
07**	-2.62/ -2.71	RASL11B	XM_ 848847	macrophage activation
**08**	-2.73	DUSP1	XM_ 002916919	regulate cytokine expression, attract phagocytic cell to inflammation site
**09**	-2.84	RAB8A	XM_ 002912702	protein localization & transport, exocytosis
**10**	-3.22	RPL30	AB 070559	RNA translation
**11**	-3.23	UBTD2	XM_ 546238	anti-apoptotic activity
**12**	-3.27	CKS2	AF 506708	anti-apoptotic activity
**13**	-4.80	SRP9	XM_ 849646	protein export
14*	-4.98/ -5.89	CRIP1	XM_ 850438	T helper cytokines regulation, immune cells differentiation and proliferation
**15**	-10.04	ID1	XM_ 847117	anti-apoptotic activity, TGF-beta signaling pathway
**16**	-∞	RNF7	XM_ 003433156	anti-apoptotic activity
17	-∞	RPL39	NG_ 016250	RNA translation

# dash symbol (-) represents down regulation of genes in order to differentiate with fold change values of up regulated genes.

**Two transcripts from the same gene with similar E-value, score and gaps.

### Real-time RT-qPCR analysis of CRFK infected cells

Three host genes (A3H, PD-1, and PD-L1) were selected for real-time RT-qPCR analysis, because they were highly up-regulated and may play important roles during FIPV induced disease; judging by their functions. A3H was involved in viral RNA and DNA editing, causing mutation, while PD-1 and its ligand (PD-L1) were involved in programmed cell death that was associated with negative regulation of immune response. Comparisons of fold change results of real-time PCR and transcriptome study for A3H and PD-L1 genes revealed almost similar levels of fold changes. The transcriptome resulted in 4.07 and 5.65 fold change for 2 transcripts of A3H while real-time PCR resulted in 5.23 ± 1.15 fold changes (Tables 1 and 3). Meanwhile, transcriptome resulted in 3.29 and 4.24 fold changes for 2 transcripts of PD-L1 gene while real-time PCR resulted in 3.97 ± 0.29 fold changes. In the case of the PD-1 gene, RNA sequencing was unable to detect the gene expression, due to low coverage (i.e., data not shown), although real-time PCR was able to detect an up-regulation of the gene at 3 h.p.i.

**Table 3:** Fold changes of A3H, PD-1 and PD-L1 genes in CRFK cells at different time points following infection with FIPV

Fold change ± SEM^*,**^			
**Time (Hours)**	**A3H**	**PD-1**	**PD-L1**
**3**	5.23 ± 1.15	8.50 ± 1.06	3.97 ± 0.29
**6**	-0.37 ± 0.03	-0.19 ± 0.01	0.97 ± 0.07
**9**	-0.41 ± 0.11	-0.23 ± 0.08	-0.21 ± 0.02
**12**	-0.23 ± 0.03	2.51 ± 0.27	-0.50 ± 0.07
**24**	-0.09 ± 0.01	-0.38 ± 0.03	-0.46 ± 0.05
**48**	991.94 ± 113.62	-0.24 ± 0.02	-0.13 ± 0.03

*GAPDH and YWHAZ as reference genes.

**Three replicates of each reaction were performed.

FIPV induced high and exceptionally high A3H expression at 3 and 48 h.p.i. respectively, but from 6 to 24 h.p.i., the gene was down-regulated. FIPV infected cells also showed high up-regulation of PD-1 expression at 3 h.p.i. and moderately up-regulated at 12 h.p.i but were being down-regulated at 6, 9, 24 and 48 h.p.i. Meanwhile, PD-L1 gene was consistently down-regulated from 3 hours to 48 h.p.i.

### Fold change expression analysis of PBMCs of FIP diagnosed cats

Peripheral Blood Mononuclear Cells (PBMCs), obtained from cats with clinical signs associated with FIP (Table 4), were purified and analysed with real-time PCR. In general, all of the FIP diagnosed cats expressed the PD-1 and PD-L1 genes more than 2 folds, while only 2 cats expressed A3H gene more than 2 folds (Table 5). The highest expression fold for A3H, PD-1 and PD-L1 genes was detected from cat no. 3 at 3.44 ± 0.36, 68.13 ± 19.45, and 96.94 ± 21.54 fold changes, respectively. Meanwhile, cat no. 5 showed less fold changes of the genes compared to the other FIP diagnosed cats.

**Table 4:** Clinical and serological parameters of FIPV diagnosed and normal cats used in this study

**Cat ID**	**Age**	**Form of FIP**	FCoV Ab**	**FeLV Ab**	**FIV Ab**
1	adult	wet form: abdominal effusion	S4	(-)	(-)
2	8 months	wet form: abdominal effusion	S3	(-)	(-)
3	1 year	wet form: pleural effusion	S4	(-)	(-)
4	3 years	wet form: abdominal effusion	S4	(-)	(-)
5	8 months	wet form: abdominal effusion	S4	(-)	(-)
6	1 year	wet form: pleural effusion	S5	(-)	(-)
7*	3 years	negative control, healthy	S1	(-)	(-)
8*	7 years	negative control, healthy	S1	(-)	(-)
9*	1 year	negative control, non-healing wound	S1	(-)	(-)
10*	1 year	negative control, healthy	S1	(-)	(-)
11*	7 months	negative control, healthy	S1	(-)	(-)

FCoV = feline coronavirus, FIV = feline immunodeficiency virus, FeLV = feline leukemia virus.

*Normal cat as control.

**The kit’s CombScale range from S0 to S6. Only cats with titer S3-S6 are considered to be positive for FIP based on the manual. The manual stated that S3, a significant positive response is equivalent to IFA titer ≥1:20.

**Table 5:** Fold changes of A3H, PD-1 and PD-L1 genes from FIP diagnosed cats

Fold change ± SEM^*,**^			
**Cat ID**	**A3H**	**PD-1**	**PD-L1**
**1**	1.11 ± 0.14	7.29 ± 1.56	18.88 ± 3.67
**2**	1.51 ± 0.16	35.26 ± 6.59	20.79 ± 3.19
**3**	3.44 ± 0.36	68.13 ± 19.45	96.94 ± 21.54
**4**	1.36 ± 0.11	40.73 ± 5.50	75.63 ± 3.73
**5**	1.27 ± 0.08	2.49 ± 0.48	3.04 ± 0.23
**6**	2.70 ± 0.35	28.73 ± 6.30	45.50 ± 4.27

*YWHAZ as sole reference gene.

**Three replicates of each reaction were performed.

## Discussion

The pathogenesis of feline coronavirus infection is unclear. The reference feline genome sequence assembly of transcriptome analysis of early infection (3 h.p.i.) of CRFK cells with FIPV 79–1146 showed that the expressions of 215 transcripts (0.8% of the trimmed annotated) were statistically significant, based on Kal’s Z test. Only 96 transcripts, which consisted of 44 up-regulated genes and 17 down-regulated genes, were expressed with fold absolute changes of 2 or more. Since only one sample per group was analysed, Kal’s Z test was used to determine the significant differences in the expression profiles. Study has shown that this test evaluates single sample against another single sample where each group in an experiment has only one sample [16]. This test is based on an approximation of the binomial distribution by the normal distribution considering proportions rather than raw counts so that it can be used reliably on libraries of different size.

The transcriptional profiles of selected genes in FIPV *in vitro* infected cells, as well as cats diagnosed with FIP, were explored. The expressions of A3H, which involved in viral RNA and DNA editing causing mutation during RNA virus infection [17] and PD-1 and its ligand (PD-L1), which are involved in programmed cell death and negative regulation of T cells immune response [18], were characterised. A3H has antiretroviral activity by generating lethal hypermutations in viral genomes and is associated with increased resistance to HIV-1 infection in certain populations [19]. In the case of felines, A3H (but not A3C) has been found to reduce the infectivity of feline leukemia virus [17]. It is interesting to detect that the expression of A3H is readily expressed at higher levels in PBMCs than in FIPV infected cells (Tables 3 and 5), indicating the possible involvement of the gene in antiviral activity. Furthermore, the up-regulation of the gene is less, compared to PD-1 and PD-L1 in FIPV diagnosed cats. In addition, the expressions of A3H are significantly up-regulated in FIPV infected CRFK cells at 48 h.p.i. The actual reason for this high expression of A3H is unclear, but suggests that this gene is essential in restricting viral replication or forming part of the type 1 interferon-induced innate response, since recent study has indicated the involvement of A3H in restricting virus replication [17,20].

Our results show that up-regulation of PD-1 and PD-L1 gene’s expression in PBMCs occurred in cats diagnosed with the FIP disease. In general, their expressions are correlated to each other. Similar patterns were also observed in chronic FIV infection [21] and in HIV infection in humans [22]; where they were associated with increasing immune dysfunction and T cell depletion. Previous studies have shown that although FIPV cannot infect CD4+ and CD8+ T-cells [8], cats infected with the virus showed T cell depletion by apoptosis resulting in an acute immunodeficiency [7]. Hence, we hypothesized that PD-L1 could be a mediator that mediates apoptosis of CD4+ and CD8+ T-cells, since its expression is found in a wide range of nonhematopoietic cells [23]. Furthermore, the blockade of PD-L1 expression was found to enhance T cell immunity and cytokine production [24]. Nevertheless, more studies are required to confirm our hypothesis.

It has been established that cats infected with FIPV undergo extensive tissue destruction due to inflammatory reaction [2]. Even though the study was performed on infected CRFK cells, a kidney epithelial cells, it is interesting to note that transcriptome analysis of cells proposed that the inflammation process was associated with proinflammatory and Th1-like cytokines production, due to the up-regulation of several chemokine genes, such as CCL8, CXCL10, and CCL17; and genes associated with innate immune responses, such as PHF11, ATF3, and IRF1. Further study on infection of FIPV in particularly type I FIPV on macrophage-like cells namely fcwf-4 cells and samples from FIP diagnosed cats will add more value to our findings. Furthermore, the down-regulation of CRIP1 (a T helper regulatory gene) (Table 2) also suggests that FIPV infection is associated with Th1 response, based on a study on CRIP1 gene in mice, transgenic mice, and in murine cell line [25]. In that study, they found that the down-regulation of CRIP was associated with the expression of IL-2, IFN-γ, and TNF-α. Previous studies have shown that *in vitro* and *in vivo* FIPV infections were associated with TNF-α and IFN-γ expressions [9,26].

Based on previous studies on the growth of FIPV 79–1146 in CRFK cells, production of progeny virus start between 3 and 6 hours post inoculation and increased rapidly until 12 hours post inoculation [27]. In our study, we found that at 3 hours, a few complete virus genomes that completely aligned to FIPV 79–1146 reference genome sequence (data not shown) has already been assembled. MX1 expression was up-regulated in FIPV infection (Table 1), similar to other RNA virus infections, its role in FIP pathogenesis is still unclear and requires further investigation. Previous studies have shown that the expression of MX1 gene inhibits viral replication during various RNA virus infections [28,29]. Early *in vitro* FIPV infection is also associated with a marked increase in the expression of RSAD2 (radical S-adenosyl methionine domain-containing protein 2) also known as viperin (Table 1). Previous studies have shown viperin involvement in inhibiting viral RNA and/or protein synthesis during different virus infections, such as the West Nile virus, Dengue virus, and hepatitis virus [30,31]. Hence, further study is required to define the role of viperin in FIPV replication and infection. Serine Proteinase Inhibitor clade B member 1 (SERPINB1) is the only gene that was markedly down-regulated (Table 2). SERPINB1 functions as an inhibitor of the neutrophil serine proteases, found at inflammatory sites where the inhibition of the gene prevents tissue destruction by phagocytic cells during the virus clearance process by infiltrating neutrophils and monocytes [32,33]. Thus, SERPINB1 down-regulation could possibly be part of innate immune response in recruiting phagocytic cells for the proteolytic destruction of infected cells. Besides genes that modulate T cell functions, several genes with pro-apoptotic and/or anti-apoptotic are also differentially regulated; thus highlighting their functions in regulating the apoptosis of virus infected cells (Additional file 1: Table S1 and Additional file 1: Table S2). However, the pro-apoptotic gene YAP-65 or YAP-1, which was found up-regulated in FIV infection in CRFK cells [34], was not up-regulated in this study.

## Conclusion

In conclusion, this present study has described the transcriptional profiles of cellular genes in vitro system can be applied to an in vivo situation and the possible involvement of A3H, PD-1, and PD-L1 genes during FIPV infection. However, further studies are required to elucidate the roles of these genes, and their interactions with other genes, during FIPV replication and infection especially in *in vivo* model.

## Materials and methods

### Virus and cell lines

Monolayers of Crandell Rees Feline Kidney (CRFK) cells (ATCC® no. CCL-94™) were grown in a base media consisting of Minimum Essential Medium (MEM), 10% Fetal Bovine Serum (FBS), 2% penicillin and streptomycin, 2% amphotericin B, and non-essential amino acids at 37°C and 5% CO2. For transcriptome study, CRFK cells were infected with FIPV 79–1146 (ATCC® no. VR-2126™) at a Multiplicity Of Infection (MOI) of 2. The virus was incubated for one hour for virus attachment. After incubation, 1% FBS MEM was added and the cells were incubated for a further 3 hours. At the end of incubation, the cells were harvested using TrypLE™ and centrifuged twice in a PBS at 4°C for 5 minutes at 1000rpm. Cell pellets were stored at -80°C until RNA extraction. For the control sample, the same process was applied, with the exception that 2 ml of sterile PBS was used to replace the virus.

### RNA extraction and sequencing

An RNeasy® Mini Kit (Qiagen®, USA) was used to extract and purify RNA samples as per the methods recommended by the manufacturer. The quality of the extracted RNA was determined by an Ultrospec 3000 Pro UV/Visible spectrophotometer (GE Healthcare, UK), where samples with an absorbance ratio value (A260/A280) of 1.8 to 2.0 were considered for further analysis with an Agilent® 2100 Bioanalyzer. Samples with RNA Integrity Numbers (RIN) 9 to 10, and concentrations higher than 500 ng/μl per sample, were sent for Illumina GAII sequence analysis.

### Bioinformatics analysis

A total of 17.3 Gb of sequencing data, comprised of both control and infected samples, was imported into the CLC bio Genomic Workbench (GWB). The sequences were trimmed for adapter sequences and low quality base. The trimmed raw sequences were subjected to RNA-sequence analysis, by mapping them to an annotated feline genome reference sequence [35] accounting for a maximum of two gaps or mismatches in each sequence. Unpaired group comparisons, based on Reads Per Kilo base per Million (RPKM) [36], were chosen as expression values for comparison. Kal’s Z test statistical analyses, based on False Discovery Rate (FDR) <0.05 and fold change >1.99 were used to filter the expressed transcripts. The resulting list was then BLAST at NCBI servers (http://www.ncbi.nlm.nih.gov/) using GWB’s built-in BLAST (blastn, refseq_rna or nr databases, mammals only). Homologous sequences with the lowest e-value, highest score, and lowest percentage of gaps to the query sequence, was selected as the transcript identity.

### Real-time RT-qPCR analysis of FIPV infected CRFK cells

In order to validate the transcriptome results, the expression profiles of 3 genes (A3H, PD-1, and PD-L1) were analysed using real-time PCR. Briefly, viral RNA from FIPV strain 79–1146 infected CRFK cells at 3, 6, 9, 12, 24, and 48 hours post infection (h.p.i.) were collected and processed as described previously. Control cells, inoculated with PBS only, were used as a control. Primers were designed using Primer-BLAST (http://www.ncbi.nlm.nih.gov/tools/primer-blast/) and synthesized by AITbiotech PTE LTD (Singapore) (Table 6).

**Table 6:** Primers sequences used in this study

**Target gene**	**Accession number**	**Sequence 5′ – 3′**	**Reference**
GAPDH	NM 001009307	F : AGTATGATTCCACCCACGGCA	[36]
		R : GATCTCGCTCCTGGAAGATGGT	
YWHAZ	EF458621	F: ACAAAGACAGCACGCTAATAATGC	[37]
		R: CTTCAGCTTCATCTCCTTGGGTAT	
PD-1	EU295528	F: GAGAACGCCACCTTCGTC	[19]
		R: TGGGCTCTCATAGATCTGCGT	
PD-L1	EU246348	F: CGATCACAGTGTCCAAGGACC	[19]
		R: TCCGCTTATAGTCAGCACCG	
A3H	EF173020	F: ACCCACAATGAATCCACTACAG	This study
		R: AGGCAGTCTTTGTGAATTAGGG	

F, forward primer, R, reverse primer.

The reactions were performed using SensiFAST™ SYBR No-ROX One Step kit (Bioline Ltd, UK) on Bio-Rad CFX 96™ Real-Time System, with C1000™ Thermal Cycler (Bio-Rad Laboratories, USA). Briefly, the reaction mixture of 20 μl contained 10 μl 2× SensiFAST SYBR No-ROX One-Step mix, 0.5 μl forward & reverse primers (5 nM for GADPH, PD-L1 and A3H, 3 nM for PD-1 and 10 nM for YWHAZ), 0.2 μl RT, 0.4 μl RiboSafe RNase inhibitor, 2.4 μl H_2_O, and 6 μl extracted RNA. The RT-qPCR reaction conditions were as follows; one cycle at 45°C for 10mins, one cycle at 95°C for 2 mins, and 35 cycles at 95°C for 5 secs; then 57°C (YWHAZ), 58°C (PD-L1), 59°C (GAPDH), 64°C (A3H), and 65°C (PD-1) for 20 secs; and finally, at 72°C for 5 secs. One cycle for the dissociation curve for all reactions was added and melting curve analysis was performed. Data generated from the technical triplicate experiment was analysed with 2^–ΔΔCT^ method [37] using Bio-Rad CFX Manager version 2.0. GAPDH and/or YWHAZ genes were chosen as reference genes, based on previous studies [38,39].

### Real-time RT-qPCR analysis of peripheral blood mononuclear cells from FIP diagnosed cats

Besides FIPV infected cell cultures, Peripheral Blood Mononuclear Cells (PBMCs) were also used to analyse the transcriptome results. Six FIP diagnosed domestic short hair breed cats, with ages ranging from 8 months to adult, that were admitted to University Veterinary Hospital, UPM, were considered for this study (Table 4). The cats tested negative for FeLV and FIV antibodies, but positive for FCoV antibodies, and showed abdominal/pleural effusion. Meanwhile, 5 healthy cats, with negative results for FCoV, FeLV, and FIV antibodies, were selected as controls. The kits for FCoV, FeLV and FIV antibody tests originated from Biogal’s feline coronavirus (FCoV) [FIP] ImmunoComb® Antibody test kit (Biogal Galed Laboratories, USA) and IDEXX’s SNAP® Combo FeLV Ag/FIV Antibody test kit (IDEXX Laboratories, USA), respectively. The tests were performed as per the methods recommended in their respective manuals. The health assessment and blood collection of the cats were performed by a trained and certified veterinarian (GTS). The sampling were performed according to internationally recognized guidelines and recommended by the Animal Care and Use Committee at the Faculty of Veterinary Medicine, Universiti Putra Malaysia.

Two to 5 ml of cat blood was drawn and stored at 4°C in BD Vacutainer® (BD USA) EDTA-K2 tubes. Parts of the blood were used for the test kits, while the rest was processed for PBMCs extraction. PBMCs were isolated using the Ficoll-Paque™ Plus (GE Healthcare, USA) method, according to the manufacturers protocol. Total RNA from PBMCs was isolated using an RNeasy mini plus kit (Qiagen, Germany), as described by the manufacturer. RNA quantity and purity was measured and assessed using a Nanodrop Nanophotometer P-class (Implen GmbH, Germany). The isolated RNA samples were either kept at -80°C for further analysis, or immediately used for real-time RT-qPCR analysis.

## Abbreviations

BLAST: Basic Local Alignment Search Tool; CRFK: Crandell Rees Feline Kidney; CMI: Cell-mediated immunity; FCOV: Feline coronavirus; FDR: False discovery rate; FECV: Feline enteric coronavirus; FIPV: Feline infectious peritonitis virus; FIV: Feline immunodeficiency virus; kb: Kilobase; min: Minute; ml: Mililitre; MOI: Multiplicity of infection; NGS: Next generation sequencing; PBMCs: Peripheral Blood Mononuclear Cells; PCR: Polymerase chain reaction; RPKM: Reads per kilobase of exon model per million mapped reads.

## Competing interests

The authors declare that we have no competing interests.

## Authors’ contributions

MSRH & COK - Cell culture works; virus inoculation; RNA extraction, purification and quantification; RT-qPCR assays; bioinformatic analysis; data analysis and interpretation; wrote the manuscript. GTS – Diagnose cats; collect blood samples from cats with FIP symptoms; perform FCoV, FIV and FeLV kit tests. TSW – helps with PCR; primers design; RT-qPCR assays and data analysis. SSA, MHB & ARO – secure & manage fundings; coordinated the project; designed experiments and collaborated in analyzing data and writing the manuscript. All the authors have read and approved the final manuscript.

## Supplementary Material

Additional file 1: Table S1 List of 76 transcripts from 44 up regulated genes with proportions fold change of 2 or more (Kal’s Z test, FDR < 0.05) with their BLAST results, NCBI accession number and gene product function. **Table S2.** List of 20 transcripts from 17 down regulated genes with proportions fold change of -2 or more (Kal’s Z test, FDR < 0.05) with their BLAST results, NCBI accession number and gene product function. Click here for file

## References

[B1] Sharif S Arshad SS Hair-Bejo M Omar AR Zeenathul NA Hafidz MA Prevalence of feline coronavirus in two cat populations in Malaysia J Feline Med Surg 2009 10 1031 1034 19818660 10.1016/j.jfms.2009.08.005 PMC7128893

[B2] Pedersen NC A review of feline infectious peritonitis virus infection: 1963–2008 J Feline Med Surg 2009 10 225 258 10.1016/j.jfms.2008.09.008 19254859 PMC7129802

[B3] Hartmann K Feline infectious peritonitis Vet Clin North Am Small Anim Pract 2005 10 1 39 79 10.1016/j.cvsm.2004.10.011 15627627 PMC7114919

[B4] Herrewegh AA Vennema H Horzinek MC Rottier PJ De Groot RJ The molecular genetics of feline coronaviruses: comparative sequence analysis of the ORF7a/7b transcription unit of different biotypes Virol 1995 10 622 631 10.1006/viro.1995.1520 PMC7131361 7571432

[B5] Vennema H Poland A Foley J Pedersen NC Feline infectious peritonitis viruses arise by mutation from endemic feline enteric coronaviruses Virol 1998 10 150 157 10.1006/viro.1998.9045 PMC7131759 9527924

[B6] Licitra BN Millet JK Regan AD Hamilton BS Rinaldi VD Duhamel GE Mutation in spike protein cleavage site and pathogenesis of feline coronavirus Emerg Infect Dis 2013 http://dx.doi.org/10.3201/eid1907.121094/10.3201/eid1907.121094 PMC3713968 23763835

[B7] De Groot Mijnes JD Van Dun JM Van Der Most RG De Groot RJ Natural history of a recurrent feline coronavirus infection and the role of cellular immunity in survival and disease J Virol 2005 10 1036 1044 10.1128/JVI.79.2.1036-1044.2005 15613332 PMC538555

[B8] Haagmans BL Egberink HF Horzinek MC Apoptosis and T-cell depletion during feline infectious peritonitis J Virol 1996 10 12 8977 8983 8971027 10.1128/jvi.70.12.8977-8983.1996 PMC190995

[B9] Takano T Hohdatsu T Hashida Y Kaneko Y Tanabe M Koyama H A “possible” involvement of TNF-alpha in apoptosis induction in peripheral blood lymphocytes of cats with feline infectious peritonitis Vet Microbiol 2007 10 2–4 121 131 17046178 10.1016/j.vetmic.2006.08.033 PMC7117258

[B10] Assarsson E Greenbaum JA Sundström M Schaffer L Hammond JA Pasquetto V Oseroff C Hendrickson RC Lefkowitz EJ Tscharke DC Sidney J Grey HM Head SR Peters B Sette A Kinetic analysis of a complete poxvirus transcriptome an immediate early class of gene Proc Natl Acad Sci U S A 2008 10 6 2140 2145 10.1073/pnas.0711573105 18245380 PMC2542872

[B11] Nanda S Havert MB Calderón GM Thomson M Jacobson C Hepatic transcriptome analysis of hepatitis C virus infection in chimpanzees defines unique gene expression patterns associated with viral clearance PLoS ONE 2008 10 10 e3442 doi:10.1371/journal.pone.0003442 10.1371/journal.pone.0003442 18927617 PMC2562457

[B12] Dowling RJ Bienzle D Gene-expression changes induced by Feline immunodeficiency virus infection differ in epithelial cells and lymphocytes J Gen Virol 2005 10 8 2239 2248 10.1099/vir.0.80735-0 16033971

[B13] Ertl R Birzele F Hildebrandt T Klein D Viral transcriptome analysis of feline immunodeficiency virus infected cells using second generation sequencing technology Vet Immunol Immunopathol 2011 10 3–4 314 324 21742384 10.1016/j.vetimm.2011.06.010

[B14] Zhang Z Schwartz S Wagner L Miller W A greedy algorithm for aligning DNA sequences J Comput Biol 2000 10 203 214 10.1089/10665270050081478 10890397

[B15] van Hamme E Dewerchin HL Cornelissen E Nauwynck HJ Attachment and internalization of feline infectious peritonitis virus in feline blood monocytes and Crandell feline kidney cells J Gen Virol 2007 10 2527 2532 10.1099/vir.0.82991-0 17698663

[B16] Kal AJ Van Zonneveld AJ Benes V Van Den Berg M Koerkamp MG Albermann K Strack N Ruijter JM Richter A Dujon B Ansorge W Tabak HF Dynamics of gene expression revealed by comparison of serial analysis of gene expression transcript profiles from yeast grown on two different carbon sources Mol Biol Cell 1999 10 6 1859 1872 10.1091/mbc.10.6.1859 10359602 PMC25383

[B17] Münk C Beck T Zielonka J Hotz-Wagenblatt A Chareza S Battenberg M Thielebein J Cichutek K Bravo IG O’Brien SJ Löchelt M Yuhki N Functions, structure, and read-through alternative splicing of feline APOBEC3 genes Genome Biol 2008 10 3 R48 10.1186/gb-2008-9-3-r48 18315870 PMC2397500

[B18] Shen T Chen X Chen Y Xu Q Lu F Liu S Increased PD-L1 expression and PD-L1/CD86 ratio on dendritic cells were associated with impaired dendritic cells function in HCV infection J Med Virol 2010 10 7 1152 1159 10.1002/jmv.21809 20513078

[B19] Li MM Emerman M Polymorphism in human APOBEC3H affects a phenotype dominant for subcellular localization and antiviral activity J Virol 2011 10 16 8197 8207 10.1128/JVI.00624-11 21653666 PMC3147987

[B20] Zhen A Du J Zhou X Xiong Y Yu XF Reduced APOBEC3H variant anti-viral activities are associated with altered RNA binding activities PLoS ONE 2012 10 7 e38771 doi:10.1371/journal.pone.0038771 10.1371/journal.pone.0038771 22859935 PMC3408456

[B21] Folkl A Wen X Kuczynski E Clark ME Bienzle D Feline programmed death and its ligand: characterization and changes with feline immunodeficiency virus infection Vet Immunol Immunopathol 2010 10 1–2 107 114 19931185 10.1016/j.vetimm.2009.10.019

[B22] Trautmann L Janbazian L Chomont N Said EA Gimmig S Bessette B Boulassel MR Delwart E Sepulveda H Balderas RS Routy JP Haddad EK Sekaly RP Upregulation of PD-1 expression on HIV-specific CD8+ T cells leads to reversible immune dysfunction Nat Med 2006 10 1198 1202 10.1038/nm1482 16917489

[B23] Keir ME Butte MJ Freeman GJ Sharpe AH PD-1 and its ligands in tolerance and immunity Annu Rev Immunol 2008 10 26 677 704 18173375 10.1146/annurev.immunol.26.021607.090331 PMC10637733

[B24] Brown JA Dorfman DM Ma FR Sullivan EL Munoz O Wood CR Greenfield EA Freeman GJ Blockade of programmed death-1 ligands on dendritic cells enhances T cell activation and cytokine production J Immunol 2003 10 3 1257 1266 12538684 10.4049/jimmunol.170.3.1257

[B25] Cousins RJ Foster LL Regulation of cysteine-rich intestinal protein, a zinc finger protein, by mediators of the immune response J Infect Dis 2000 10 1 S81 S84 doi:10.1086/315917 10944488 10.1086/315917

[B26] Berg AL Ekmanb K Belákb S Bergb M Cellular composition and interferon-γ expression of the local inflammatory response in feline infectious peritonitis (FIP) Vet Microbiol 2005 10 1–2 15 23 16183217 10.1016/j.vetmic.2005.07.017 PMC7117157

[B27] Dewerchin HL Cornelissen E Nauwynck HJ Replication of feline coronaviruses in peripheral blood monocytes Arch Virol 2005 10 2483 2500 10.1007/s00705-005-0598-6 16052283 PMC7086860

[B28] Khaiboullina SF Rizvanov AA Deyde VM St Jeor SC Andes virus stimulates interferon-inducible MxA protein expression in endothelial cells J Med Virol 2005 10 2 267 275 10.1002/jmv.20266 15602733

[B29] Hoenen A Liu W Kochs G Khromykh AA Mackenzie JM West Nile virus-induced cytoplasmic membrane structures provide partial protection against the interferon-induced antiviral MxA protein J Gen Virol 2007 10 11 3013 3017 10.1099/vir.0.83125-0 17947524

[B30] Jiang D Weidner JM Qing M Pan XB Guo H Xu C Zhang X Birk A Chang J Shi PY Block TM Guo JT Identification of five interferon-induced cellular proteins that inhibit west nile virus and dengue virus infections J Virol 2010 10 16 8332 8341 10.1128/JVI.02199-09 20534863 PMC2916517

[B31] Helbig KJ Lau DT Semendric L Harley HA Beard MR Analysis of ISG expression in chronic hepatitis C identifies viperin as a potential antiviral effector Hepatology 2005 10 3 702 710 10.1002/hep.20844 16108059

[B32] Gong D Benarafa C Hartshorn KL Remold-O’Donnell E The neutrophil serine protease inhibitor SerpinB1 protects against inflammatory lung injury and morbidity in influenza virus infection J Immunol 2009 10 43 10

[B33] Benarafa C LeCuyer TE Baumann M Stolley JM Cremona TP Remold-O’Donnell E SerpinB1 protects the mature neutrophil reserve in the bone marrow J Leukoc Biol 2011 10 1 21 29 10.1189/jlb.0810461 21248149 PMC3114599

[B34] Basu S Totty NF Irwin MS Sudol M Downward J Akt phosphorylates the Yes-associated protein, YAP, to induce interaction with 14-3-3 and attenuation of p73-mediated apoptosis Mol Cell 2003 10 1 11 23 10.1016/S1097-2765(02)00776-1 12535517

[B35] The UCSC felCat4 data from the Dec. 2008 catChrV17e draft assembly http://genome.ucsc.edu/goldenPath/credits. html#cat_credits

[B36] Mortazavi A Williams BA McCue K Schaeffer L Wold B Mapping and quantifying mammalian transcriptomes by RNA-Seq Nat Methods 2008 10 7 621 628 10.1038/nmeth.1226 18516045 PMC13303166

[B37] Livak K Schmittgen TD Analysis of relative gene expression data using real-time quantitative PCR and the 2(-Delta Delta C(T)) Method Methods 2001 10 4 402 408 10.1006/meth.2001.1262 11846609

[B38] Penning LC Vrieling HE Brinkhof B Riemers FM Rothuizen J Rutteman GR Hazewinkel HA A validation of 10 feline reference genes for gene expression measurements in snap-frozen tissues Vet Immunol Immunopathol 2007 10 212 222 10.1016/j.vetimm.2007.08.006 17904230

[B39] Kessler Y Helfer-Hungerbuehler AK Cattori V Meli ML Zellweger B Ossent P Riond B Reusch CE Lutz H Hofmann-Lehmann R Quantitative TaqMan® real-time PCR assays for gene expression normalisation in feline tissues BMC Mol Biol 2009 10 106 doi:10.1186/1471-2199-10-106 10.1186/1471-2199-10-106 20003366 PMC2803789

