# Supplementary material for: Transcriptional profiling of feline infectious peritonitis virus infection in CRFK cells and in PBMCs from FIP diagnosed cats
Source: Virol J. 2013 Nov 9;10:329. doi: 10.1186/1743-422X-10-329 (PMC3829811; doi:10.1186/1743-422X-10-329)
Supplement: Additional file 1: Table S1 — List of 76 transcripts from 44 up regulated genes with proportions fold change of 2 or more (Kal’s Z test, FDR < 0.05) with their BLAST results, NCBI accession number and gene product function. Table S2. List of 20 transcripts from 17 down regulated genes with proportions fold change of -2 or more (Kal’s Z test, FDR < 0.05) with their BLAST results, NCBI accession number and gene product function. [file 1743-422X-10-329-S1.docx]

**Transcriptome - Gene Expression: All Up Regulated Genes**

Table S1 - List of 76 transcripts from 44 up regulated genes with proportions fold change of 2 or more (Kal’s Z test, FDR < 0.05) with their BLAST results, NCBI accession number and gene product function.

| **No.** | **Feature ID** | **Kal’s Z Test Proportions  Fold Change** | **BLAST Result** | | **E-value (Score) [Gaps]** | **Accession Number** | **Gene Product Function** |
| --- | --- | --- | --- | --- | --- | --- | --- |
| **01** | CCL8 (ENSFCAG 00000013594) | ∞ | | monocyte chemoattractant protein-2 | 4.22E-41 (90)  [0%] | S 67956 | cytokine/chemokine - induce Th2 cytokines  attract monocyte, lymhpocyte, NK cell, dendritic cell, basophil and eosinophil |
| **02** | ENSFCAG 00000013594 | ∞ | | monocyte chemoattractant protein-2 | 4.22E-41 (90)  [0%] | S 67956 | cytokine/chemokine - induce Th2 cytokines  attract monocyte, lymhpocyte, NK cell, dendritic cell, basophil and eosinophil |
| **03** | ENSFCAG 00000000708 | 22.8 | | radical S-adenosyl methionine domain containing 2 protein | 1.24E-82 (160) [2%] | XM_ 002921192 | interferon stimulated gene  inhibit viral protein and RNA synthesis |
| **04** | RSAD2 (ENSFCAG 00000000708) | 19.33 | | radical S-adenosyl methionine domain containing 2 protein | 4.88E-82 (160) [2%] | XM_ 002921192 | interferon stimulated gene  inhibit viral protein and RNA synthesis |
| **05** | CXCL10 (ENSFCAG 00000009503) | 12.98 | | C-X-C motif chemokine 10 like | 2.41E-33 (77) [0%] | XM_ 002924730 | cytokine/chemokine - induce Th1 cells response  stimulate monocyte, eosinophil, NK cell and T cell migration |
| **06** | ENSFCAG 00000003892 | 12.73 | | SLAM member family 7 like | 9.94E-105 (198) [0%] | XM_ 002928442 | stimulate NK cell cytotoxicity, B cell growth and B cell TNF-alpha and IL-14 secretion, promote lymphocytes adhesion |
| **07** | SLAMF7 (ENSFCAG 00000003892) | 11.36 | | SLAM member family 7 like | 9.94E-105 (198) [0%] | XM_ 002928442 | stimulate NK cell cytotoxicity, B cell growth and B cell TNF-alpha and IL-14 secretion, promote lymphocytes adhesion |
| 08 | ENSFCAG 00000009503 | 7.64 | | C-X-C motif chemokine 10 like | 9.22E-34 (77)  [0%] | XM_ 002924730 | cytokine/chemokine - induce Th1 cells response  stimulate monocyte, eosinophil, NK cell and T cell migration |
| 09 | ENSFCAG 00000007838 | 6.68 | | follistatin related protein 3 like | 1.23E-95 (182) [0%] | XM_ 850129 | regulate cell to cell adhesion  inhibit cell growth and diffrerentiation |
| 10 | ATF3 (ENSFCAG 00000014611) | 6.11 | | cyclic AMP dependent activating transcription factor 3 ATF-3, liver regeneration factor 1 LRF-1 | 8.23E-94 (178) [0%] | XM_ 847382 | promote apoptosis and cell proliferation  suppres pro-inflammatory cytokines expression |

Table S1 - continued

| **No.** | **Feature ID** | **Kal’s Z Test**  **Proportions  Fold Change** | **BLAST Result** | **E-value (Score) [Gaps]** | **Accession Number** | **Gene Product Function** |
| --- | --- | --- | --- | --- | --- | --- |
| **11** | MFSD2A (ENSFCAG 00000009858) | 6.04 | major facilitator superfamily domain containing 2A | 5.04E-127 (235) [0%] | AL 663070 | regulate cell growth, cell adhesion and cell motility |
| **12** | ENSFCAG 00000000449 | 5.76 | ETS related transcription factor, Elf 3 like | 5.91E-97 (184) [0%] | XM_ 002914595 | regulate inflammation  and cell differentiation |
| **13** | ENSFCAG 00000013210 | 5.65 | antiviral cytidine deaminase A3Cc, A3Ca, A3Cb & A3Ch | 0 (488) [0%] | EU 109281 | interferon stimulated gene  edit viral RNA/DNA causing mutation |
| **14** | ENSFCAG 00000007303 | 5.35 | interferon induced with helicase C domain, IFIH 1, MDA5 | 0 (497) [0%] | NG_ 011495 | viral RNA sensor  induce cytokines and infterferons expression |
| **15** | ENSFCAG 00000013143 | 4.96 | interferon induced 35kDA protein like | 7.19E-58 (119) [0%] | XM_ 002930054 | interferon stimulated gene  inhibit viral gene transcription |
| **16** | ENSFCAG 00000009858 | 4.68 | major facilitator superfamily domain containing 2A | 4.77E-128 (235) [0%] | AL 663070 | regulate cell growth, cell adhesion and cell motility |
| **17** | IFIH1 (ENSFCAG 00000007303) | 4.48 | interferon induced with helicase C domain, IFIH 1, MDA5 | 0 (497) [0%] | NG_ 011495 | viral RNA sensor  induce cytokines and infterferons expression |
| **18** | TRIM25 (ENSFCAG 00000008031) | 4.46 | similar to tripartite motif containing 25 | 5.69E-126 (234) [0%] | XM_ 548223 | E3 ligase  ubiquitination of RIG-I and ISG15  induce type I IFN |
| **19** | ENSFCAG 00000008068 | 4.46 | myxovirus (influenza virus) resistance 1, influenza inducible protein p78 (mouse), MX 1 | 1.29E-75 (150) [0%] | NM_ 001003134 | interferon stimulated gene  induce apoptosis in infected cell  prevent viral genome transcription and replication |
| **20** | MX1 (ENSFCAG 00000008068) | 4.34 | myxovirus (influenza virus) resistance 1, influenza inducible protein p78 (mouse), MX 1 | 2.67E-76 (150) [0%] | NM_ 001003134 | interferon stimulated gene  induce apoptosis in infected cell  prevent viral genome transcription and replication |
| **21** | A8WEV0_ FELCA (ENSFCAG 00000009047) | 4.24 | programmed cell death PD-L 1 | 0 (336) [0%] | EU 246348 | negative regulation of immune response  induce IL-10 |
| **22** | PHF11 (ENSFCAG 00000013097) | 4.10 | PHD finger protein 11 | 2.49E-71(142) [0] | AL 139321 | Th1 cytokines activation  contribute to T cell activation and viability |

Table S1 – continued

| **No.** | **Feature ID** | **Kal’s Z Test**  **Proportions  Fold Change** | | **BLAST Result** | | **E-value (Score) [Gaps]** | | **Accession Number** | | **Gene Product Function** | |
| --- | --- | --- | --- | --- | --- | --- | --- | --- | --- | --- | --- |
| **23** | IFI44L (ENSFCAG 00000013210) | 4.07 | antiviral cytidine deaminase A3Cc, A3Ca, A3Cb & A3Ch | | 0 (488) [0%] | | EU 109281 | | interferon stimulated gene  edit viral RNA/DNA causing mutation | |  |
| **24** | RUNX1 (ENSFCAG 00000006761) | 3.98 | runt related transcription factor 1 | | 0 (333) [0%] | | NG_ 011402 | | regulate gene transcription  needed for T cell differentiation and function | |  |
| **25** | ENSFCAG 00000008031 | 3.90 | similar to tripartite motif containing 25 | | 1.15E-126 (234) [0%] | | XM_ 548223 | | E3 ligase  ubiquitination of RIG-I and ISG15  induce type I IFN | |  |
| **26** | ENSFCAG 00000002966 | 3.79 | class E basic helix-loop-helix protein 40 like | | 0 (709) [0%] | | XM_ 002919988 | | regulate gene transcription  regulate lymphocyte activation  regulate cell cycle and cell death | |  |
| **27** | HERC5  (ENSFCAG 00000006163) | 3.69 | HECT domain and RLD 5 (HERC 5) | | 1.68E-74(147) [0%] | | XM_ 002913599 | | E3 ligase  promote cell cycle  ubiquitination of ISG15 and RIG-I | |  |
| **28** | CCL17_FELCA (ENSFCAG 00000004694) | 3.69 | chemokine (C-C motif) ligand 17 | | 8.24E-58(118) [0%] | | NM_ 001009849 | | activate T cell development, maturation and migration  Th2 cell response | |  |
| **29** | ENSFCAG 00000000928 | 3.65 | deltex 3 like DTX3L | | 0(708) [1%] | | XM_ 002927235 | | E3 ligase  protect cell from DNA damage  involves in Notch signaling | |  |
| **30** | UBA7  (ENSFCAG 00000010680) | 3.58 | UBE 1 L protein gene | | 0(377) [1%] | | AF 294032 | | E1 activating enzyme  ubiquitnate ISG15 and RIG-I  inhibit cell growth and promote apoptosis | |  |
| **31** | ENSFCAG 00000006163 | 3.56 | HECT domain and RLD 5 (HERC 5) | | 1.15E-73(147) [0%] | | XM_ 002913599 | | E3 ligase  promote cell cycle  ubiquitination of ISG15 and RIG-I | |  |
| **32** | ENSFCAG 00000006761 | 3.54 | runt related transcription factor 1 | | 0 (333) [0%] | | NG_ 011402 | | regulate gene transcription  needed for T cell differentiation and function | |  |
| **33** | ENSFCAG 00000001294 | 3.48 | DEAD (Asp-Glu-Ala-Asp) box polypeptide 58(DDX 58), RIG-I | | 1.62E-100 (192) [0%] | | XM_ 003431561 | | interferon stimulated gene  RNA virus sensor  induce cytokines and interferons | |  |

Table S1 – continued

| **No.** | **Feature ID** | **Kal’s Z Test**  **Proportions  Fold Change** | **BLAST Result** | **E-value (Score) [Gaps]** | **Accession Number** | **Gene Product Function** |
| --- | --- | --- | --- | --- | --- | --- |
| **34** | ENSFCAG 00000010680 | 3.47 | UBE 1 L protein gene | 0 (377) [1%] | AF 294032 | E1 activating enzyme  ubiquitnate ISG15 and RIG-I  inhibit cell growth and promote apoptosis |
| **35** | BAK1 (ENSFCAG 00000013657) | 3.46 | BCL-2 antagonist/killer 1 gene | 5.66E-64 (128) [0%] | DQ 926868 | induce apoptosis |
| **36** | OAS1  (ENSFCAG 00000000318) | 3.43 | 2’,5’-oligoadenylate synthetase 1 40/46 kDa, OAS 1 | 1.01E-66 (133) [0%] | NM_ 001048131 | interferon stimulated gene  induce RNase-L for viral RNA degradation |
| **37** | ENSFCAG 00000002145 | 3.37 | DEXH (Asp-Glu-X-His) box polypeptide 58 transcript variant 3 (DHX 58), LGP2 | 5.69E-86 (166) [0%] | XM_ 855439 | negative regulator of RIG-I and MDA5  promote T-cell action |
| **38** | ENSFCAG 00000009860 | 3.32 | transporter 1 (TAP1), ATP-binding cassette sub-family B | 8.22E-82 (159) [1%] | NG_ 011759 | peptide transport for MHC class I molecule |
| **39** | ENSFCAG 00000013097 | 3.31 | PHD finger protein 11 | 2.49E-71(142) [0] | AL 139321 | Th1 cytokines activation  contribute to T cell activation and viability |
| **40** | ENSFCAG 00000009047 | 3.29 | programmed cell death PD-L 1 | 0 (336) [0%] | EU 246348 | negative regulation of immune response  induce IL-10 |
| **41** | ENSFCAG 00000000318 | 3.26 | 2’,5’-oligoadenylate synthetase 1 40/46 kDa, OAS 1 | 2.80E-66 (133) [0%] | NM_ 001048131 | interferon stimulated gene  induce RNase-L for viral RNA degradation |
| **42** | DDX58 (ENSFCAG 00000001294) | 3.21 | DEAD (Asp-Glu-Ala-Asp) box polypeptide 58, DDX 58 | 5.71E-102 (192) [0%] | XM_ 003431561 | interferon stimulated gene  RNA virus sensor  induce cytokines and interferons |
| **43** | DHX58 (ENSFCAG 00000002145) | 3.07 | DEXH (Asp-Glu-X-His) box polypeptide 58 transcript variant 3, DHX 58 | 5.69E-86 (166) [0%] | XM_ 855439 | negative regulator of RIG-I and MDA5  promote T-cell action |
| **44** | ENSFCAG 00000013318 | 3.02 | interferon regulatory factor 1 IRF 1 | 0 (337) [3%] | NG_ 011450 | regulate type I IFNs expression  regulate immuna activation, apoptosis and inflammation |
| **45** | IFI44 (ENSFCAG 00000012159) | 3.02 | similar to interferon induced hepatitis C associated microtubular aggregat | 7.13E-44 (92) [0%] | XM_ 547319 | arrest cell cycle |

Table S1 - continued

| **No.** | **Feature ID** | **Kal’s Z Test**  **Proportions  Fold Change** | **BLAST Result** | **E-value (Score) [Gaps]** | **Accession Number** | **Gene Product Function** |
| --- | --- | --- | --- | --- | --- | --- |
| **46** | IFI35 (ENSFCAG 00000013143) | 3.00 | interferon induced 35 kDa protein like | 7.19E-58 (119) [0%] | XM_ 002930054 | interferon stimulated gene  inhibit viral gene transcription |
| **47** | RASSF4 (ENSFCAG 00000003482) | 2.94 | protein DEPP like | 2.04E-171 (309) [0%] | XM_ 534951 | probably contribute to cellular stress response |
| **48** | ENSFCAG 00000001009 | 2.93 | bone marrow stromal antigen 2 MYA-1 tetherin | 1.3E-163 (296) [0%] | NM_ 001243085 | prevent virion release from infected cells |
| **49** | IRF1 (ENSFCAG 00000013318) | 2.91 | interferon regulatory factor 1 IRF 1 | 0 (337) [3%] | NG_ 011450 | regulate type I IFNs expression  regulate immuna activation, apoptosis and inflammation |
| **50** | MAT2B (ENSFCAG 00000009025) | 2.42 | methionine adenosyltransferase II beta transcript variant 2 | 2.73E-90 (173) [0%] | XM_ 002926780 | regulate growth and apoptosis |
| **51** | SAMHD1 (ENSFCAG 00000012963) | 2.42 | uncharacterized protein, chromosome 20 open reading frame 111 | 3.05E-101 (193) [0%] | NM_ 016470 | cause viral RNA and protein degradation  possibly involves in negative regulation of immune system |
| **52** | BST2 (ENSFCAG 00000001009) | 2.88 | bone marrow stromal antigen 2 MYA-1 tetherin | 3.16E-164 (296) [0%] | NM_ 001243085 | prevent virion release from infected cells |
| **53** | BHLHE40 (ENSFCAG 00000002966) | 2.85 | class E basic helix-loop-helix protein 40 like | 0 (709) [0%] | XM_ 002919988 | regulate gene transcription  regulate lymphocyte activation  regulate cell cycle and cell death |
| **54** | RNF19B (ENSFCAG 00000003581) | 2.80 | ring finger protein 19B | 0 (328) [1%] | XM_ 002921875 | E3 ligase  involves in NK cell and T cell cytotoxicity |
| **55** | ENSFCAG 00000003581 | 2.72 | ring finger protein 19B | 0 (328) [1%] | XM_ 002921875 | E3 ligase  involves in NK cell and T cell cytotoxicity |
| **56** | ENSFCAG 00000013657 | 2.71 | BCL-2 antagonist/killer 1 gene | 1.95E-63 (128) [0%] | DQ 926868 | induce apoptosis |
| **57** | ENSFCAG 00000002912 | 2.71 | BTG family, member 3, transcript variant 2 | 2.26E-122 (227) [0%] | XM_ 544819 | inhibit cell cycle |

Table S1 - continued

| **No.** | **Feature ID** | **Kal’s Z Test**  **Proportions  Fold Change** | **BLAST Result** | **E-value (Score) [Gaps]** | **Accession Number** | **Gene Product Function** |
| --- | --- | --- | --- | --- | --- | --- |
| **58** | MYD88 (ENSFCAG 00000012873) | 2.62 | myeloid differentiation primary response protein MyD88 like | 1.32E-124 (230) [0%] | XM_ 002914648 | regulate innate and adaptive immune response  Toll-like receptors mediators |
| **59** | ENSFCAG 00000012963 | 2.54 | uncharacterized protein, chromosome 20 open reading frame 111 | 3.05E-101 (193) [0%] | NM_ 016470 | cause viral RNA and protein degradation  possibly involves in negative regulation of immune system |
| **60** | TAP1 (ENSFCAG 00000009860) | 2.47 | transporter 1, ATP-binding cassette, sub-family B (MDR/TAP) | 8.22E-82 (159) [1%] | NG_ 011759 | peptide transport for MHC class I molecule |
| **61** | ENSFCAG 00000001273 | 2.46 | diphtheria toxin receptor DTR, heparin binding epidermal growth factor like growth factor | 3.8E-139 (255) [1%] | AY 164533 | positive regulation of cell cycle & cell migration  inhibit apoptosis  has antiinflammaotry effects |
| **62** | ENSFCAG 00000012873 | 2.39 | myeloid differentiation primary response protein MyD88 like | 1.32E-124 (230) [0%] | XM_ 002914648 | regulate innate and adaptive immune response  Toll-like receptors mediators |
| **63** | STAT2  (ENSFCAG 00000012023) | 2.38 | apolipoprotein F | 5.1E-158 (288) [1%] | XM_ 003431467 | antiviral genes regulation  chemokine genes expression |
| **64** | ENSFCAG 00000010232 | 2.36 | alpha-1 antiproteinase antitrypsin like | 0 (356) [0%] | XM_ 002920473 | inactivate viral protease  inhibit apoptosis |
| **65** | Q005V0_FELCA (ENSFCAG 00000011831) | 2.35 | caspase 7 CASP 7 | 7.72E-94 (179) [0%] | DQ 926882 | induce apoptosis |
| **66** | ENSFCAG 00000014611 | 2.34 | cyclic AMP dependent activating transcription factor 3 ATF-3, | 2.83E-93 (178) [0%] | XM_ 847382 | promote apoptosis and cell proliferation  suppres pro-inflammatory cytokines expression |
| **67** | HBEGF (ENSFCAG 00000001273) | 2.28 | diphtheria toxin receptor DTR, heparin binding epidermal growth factor like growth factor | 5.98E-139 (255) [1%] | AY 164533 | positive regulation of cell cycle & cell migration  inhibit apoptosis  has antiinflammaotry effects |
| **68** | ENSFCAG 00000012882 | 2.22 | alpha/beta hydrolase domain containing protein 2 like | 5.38E-90 (172) [0%] | XM_ 844639 | possible needed for monocyte to macrophage differentiation |

Table S1 – continued

| **No.** | **Feature ID** | **Kal’s Z Test**  **Proportions  Fold Change** | **BLAST Result** | **E-value (Score) [Gaps]** | **Accession Number** | **Gene Product Function** |
| --- | --- | --- | --- | --- | --- | --- |
| **69** | ENSFCAG 00000012023 | 2.21 | apolipoprotein F | 5.1E-158 (288) [1%] | XM_ 003431467 | antiviral genes regulation  chemokine genes expression |
| **70** | PLIN2 (ENSFCAG 00000002659) | 2.14 | perilipin 2 | 6.43E-116 (216) [0%] | XM_ 002918522 | induce lipid uptake, accumulation and storage |
| **71** | ENSFCAG 00000004938 | 2.13 | tenascin C | 0 (667) [0%] | NM_ 001195149 | inhibit T cel activation and migration |
| **72** | ENSFCAG 00000010155 | 2.12 | integrin alpha 5, fibronectin receptor alpha polypeptide | 2.59E-63 (128) [0%] | XM_ 002923444 | involves in cell adhesion. cell signaling, T cell/monocyte activation and migration |
| **73** | ENSFCAG 00000001452 | 2.05 | similar to solute carrier family 39, zinc transporter member 14 transcript variant 3 | 9.27E-92 (176) [0%] | XM_ 543250 | transport zinc and iron |
| **74** | ABHD2 (ENSFCAG 00000012882) | 2.01 | alpha/beta hydrolase domain containing protein 2 like | 3.12E-88 (172) [0%] | XM_ 844639 | possible needed for monocyte to macrophage differentiation |
| **75** | TNC  (ENSFCAG 00000004938) | 2.01 | tenascin C | 0 (667) [0%] | NM_ 001195149 | inhibit T cel activation and migration |
| **76** | BTG3  (ENSFCAG 00000002912) | 2.00 | BTG family, member 3, transcript variant 2 | 1.29E-121 (227) [0%] | XM_ 544819 | inhibit cell cycle |

**Transcriptome - Gene Expression: All Down Regulated Genes**

Table S2 - List of 20 transcripts from 17 down regulated genes with proportions fold change of -2 or more (Kal’s Z test, FDR < 0.05) with their BLAST results, NCBI accession number and gene product function.

| **No.** | **Feature ID** | **Kal’s Z Test**  **Proportions  Fold Change** | **BLAST Result** | **E-value (Score) [Gaps]** | **Accession Number** | **Gene Product Function** |
| --- | --- | --- | --- | --- | --- | --- |
| **01** | ENSFCAG 00000002195 | -2.04 | jagged 1 JAG 1 | 0 (330) [0%] | NG_ 007496 | Toll-like receptors (TLRs) response, positive regulation of Notch signaling pathway |
| **02** | ENSFCAG 00000015502 | -2.04 | v-kit Hardy-Zuckerman 4 feline sarcoma viral oncogene homolog KIT | 1.23E-48 (102) [0%] | NM_ 001009837 | signal transduction, apoptosis inducer, clathrin dependent endocytosis |
| **03** | JUB (ENSFCAG 00000006641) | -2.14 | ajuba like protein | 0 (801) [0%] | XM_ 537368 | co-trancriptional repressor with GFI-1, cell adhesion |
| **04** | ENSFCAG 00000008689 | -2.22 | monocyte/ neutrophil elastase inhibitor gene | 2.71E-110 (206) [1%] | AF 053630 | neutrophil proteolytic activity inhibitor |
| **05** | CD59 (ENSFCAG 00000010138) | -2.34 | growth factor independent 1 transcription repressor GFI 1 | 8E-37 (165) [2%] | NM_ 001112709 | T-cell differentiation, gene transcription repression |
| **06** | ENSFCAG 00000010828 | -2.40 | coactosin like 1 | 8.26E-59 (121) [0%] | XM_ 001144958 | pro-inflammatory leukotrienes activation |
| **07** | ENSFCAG 00000006641 | -2.44 | ajuba like protein | 0 (801) [0%] | XM_ 537368 | co-trancriptional repressor with GFI-1, cell adhesion |
| **08** | RASL11B (ENSFCAG 00000007894) | -2.62 | RAS-like family 11 member B | 0 (396) [0%] | XM_ 848847 | macrophage activation |
| **09** | ENSFCAG 00000007894 | -2.71 | RAS-like family 11 member B | 0 (396) [0%] | XM_ 848847 | macrophage activation |
| **10** | ENSFCAG 00000012521 | -2.73 | dual specificity protein phosphatase 1 DUSP 1 | 2.53E-179 (321) [0%] | XM_ 002916919 | MAPK signaling pathway, regulate cytokine expression, attract phagocytic cell to inflammation site |

* dash symbol (-) represents down regulation of genes in order to differentiate with fold change values of up regulated genes.

Table S2 - continued

| **No.** | **Feature ID** | **Kal’s Z Test**  **Proportions  Fold Change** | **BLAST Result** | **E-value (Score) [Gaps]** | **Accession Number** | **Gene Product Function** |
| --- | --- | --- | --- | --- | --- | --- |
| **11** | ENSFCAG 00000009000 | -2.84 | RAS-related protein RAB-8A-like | 1.07E-58 (121) [0%] | XM_ 002912702 | protein localization & transport, exocytosis |
| **12** | ENSFCAG 00000002165 | -3.22 | RPL 30 gene for ribosomal protein L30 | 1.88E-60 (122) [1%] | AB 070559 | RNA translation |
| **13** | UBTD2 (ENSFCAG 00000000435) | -3.23 | dendritic cell derived ubiquitin like protein, ubiquitin domain containing protein 2 like | 0 (355) [0%] | XM_ 546238 | anti-apoptotic activity |
| **14** | ENSFCAG 00000004213 | -3.27 | CDC 28 protein kinase 2 CKS 2 gene | 2.3E-48 (103) [1%] | AF 506708 | anti-apoptotic activity |
| **15** | ENSFCAG 00000010275 | -4.80 | similar to signal recognition particle 9 kDa protein SRP 9 | 4.23E-54  (112) [0%] | XM_ 849646 | protein export |
| **16** | ENSFCAG 00000016258 | -4.98 | similar to cysteine rich protein 1, cysteine rich intestinal protein CRIP | 1.66E-35 (80) [0%] | XM_ 850438 | T helper cytokines regulation, immune cells differentiation and proliferation |
| **17** | CRIP1 (ENSFCAG 00000016258) | -5.89 | similar to cysteine rich protein 1, cysteine rich intestinal protein CRIP | 2.18E-35 (80) [0%] | XM_ 850438 | T helper cytokines regulation, immune cells differentiation and proliferation |
| **18** | ENSFCAG 00000004581 | -10.04 | inhibitor of DNA binding 1, dominant negative helix-loop-helix protein ID 1 | 0 (368) [0%] | XM_ 847117 | anti-apoptotic activity, TGF-beta signaling pathway |
| **19** | ENSFCAG 00000010126 | -∞ | ring finger protein 7 transcript variant 1, RNF 7 | 9.45E-80 (155) [0%] | XM_ 003433156 | anti-apoptotic activity |
| **20** | RPL39 (ENSFCAG 00000008251) | -∞ | ribosomal protein L 39 RPL 39 | 2.08E-52 (109) [0%] | NG_ 016250 | RNA translation |
